# Flipping the Switch: Innovations in Inducible Probes for Protein Profiling

**DOI:** 10.1021/acschembio.1c00572

**Published:** 2021-11-15

**Authors:** Sean M. McKenna, Ellen M. Fay, Joanna F. McGouran

**Affiliations:** † School of Chemistry and Trinity Biomedical Sciences Institute, Trinity College Dublin, 152-160 Pearse St, Dublin 2, Ireland; ‡ Synthesis and Solid State Pharmaceutical Centre (SSPC), Bernal Institute, Limerick V94 T9PX, Ireland

## Abstract

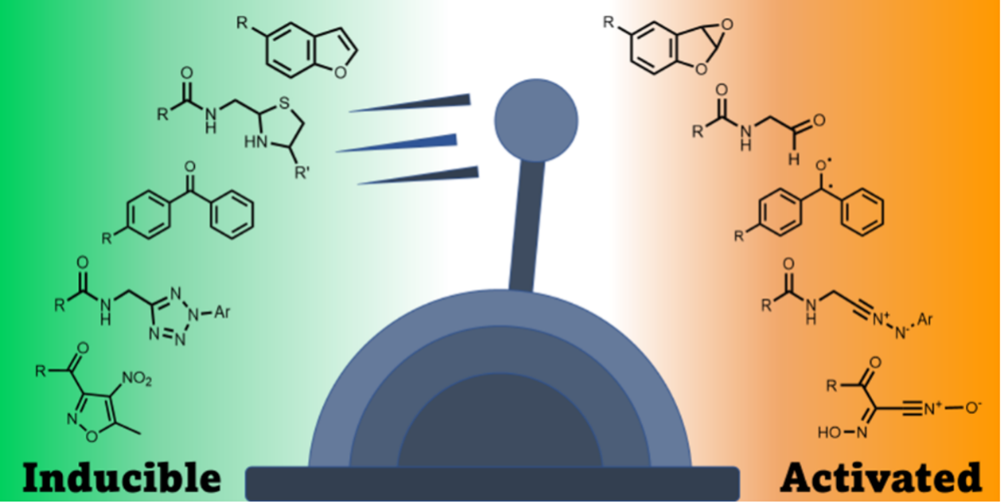

Over the past two decades, activity-based probes have enabled a range of discoveries, including the characterization of new enzymes and drug targets. However, their suitability in some labeling experiments can be limited by nonspecific reactivity, poor membrane permeability, or high toxicity. One method for overcoming these issues is through the development of “inducible” activity-based probes. These probes are added to samples in an unreactive state and require *in situ* transformation to their active form before labeling can occur. In this Review, we discuss a variety of approaches to inducible activity-based probe design, different means of probe activation, and the advancements that have resulted from these applications. Additionally, we highlight recent developments which may provide opportunities for future inducible activity-based probe innovations.

## Introduction

**Figure 1:**
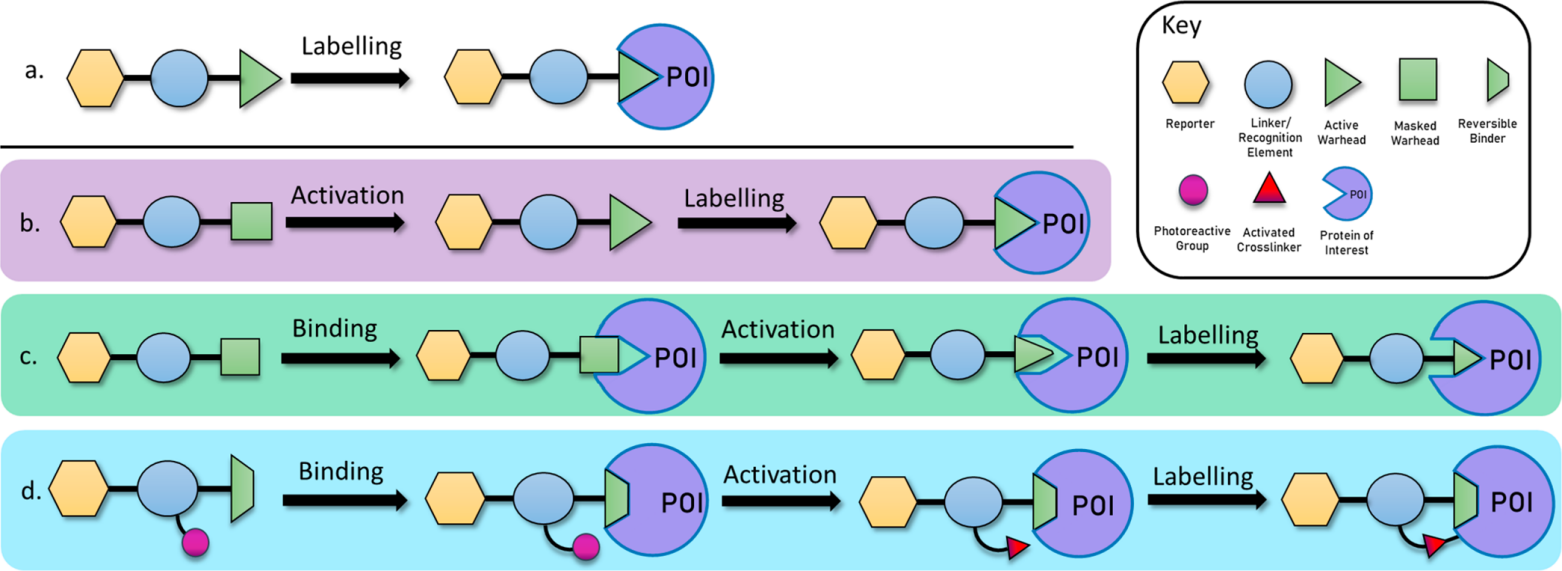
General scheme for activity-based probe labeling. (a) Traditional activity-based probe labeling. (b) Inducible activity-based probe labeling with a masked (unreactive) warhead which undergoes activation prior to labeling. (c) Inducible activity-based probe labeling with a warhead activated following binding to the protein of interest. (d) Affinity-based probe bearing a reversible binding motif and inducible cross-linking group.

Activity-based probes have been developed as effective tools for identifying active enzymes in biological samples.^1^ They enable the detection and characterization of target proteins through the formation of a covalent bond between the probe and an amino acid residue at the protein active site.^2^ This labeling reaction occurs via the “warhead” unit of the probe, commonly an electrophile, which is tethered through a linker to a detectable reporter group (Figure 1a). The linker will typically incorporate a recognition motif for the target protein. The reporter is usually a fluorescent group or an affinity tag.^3^ The reporter group may be present during labeling or can be added in a subsequent step using a bioorthogonal reaction such as copper-catalyzed azide–alkyne cycloaddition, strain-promoted azide–alkyne cycloaddition, inverse electron demand Diels–Alder tetrazine ligation, and Staudinger–Bertozzi ligation.^4−7^ Applications of probes have enabled extensive activity-based protein profiling of many cell types, leading to the characterization of novel drug targets,^8^ enzyme inhibitors,^9^ and even the discovery of new enzymatic activity.^10^

While activity-based probes have been used to probe a diverse range of active enzymes,^9,11−13^ applications of these tool compounds can have limitations. Highly electrophilic warheads lead to nonspecific protein labeling due to off-target reactivity.^14^ Activity-based probes can exhibit poor membrane permeability, preventing efficient labeling in whole cells.^15^ Many probes are also not suitable for labeling in living systems due to toxicity.^16,17^ Finally, the active site of some target proteins, including metalloenzymes, aspartyl proteases, histone deacetylases, and kinases lack a nucleophilic amino acid residue, making such proteins unsuitable for covalent capture by an electrophilic warhead.^1,18,19^ In these contexts, traditional activity-based probe approaches may be unsuitable, requiring alternate methods to be developed.

One approach for overcoming these issues has been the development of probes, which are introduced to a biological sample in an inert state and require conversion to form the reactive probe. These “inducible” probes are initially unreactive due to chemical or steric masking of the reactive warhead. Upon activation, the inducible probe is converted into a reactive state, allowing labeling to occur. This can occur prior to or following binding to the target enzyme (pre- or post-binding activation, Figure 1b–d). Inducible probes can further be grouped by their mode of activation; those that are “exogenously” induced by an external source such as UV light or a chemical reagent and those that are “endogenously” induced either by an agent found naturally in the biological sample or within the target enzyme.

## Exogenous Induction

Probes activated by exogenous induction require an outside stimulus such as UV light or a secondary reagent. UV irradiation has been used to induce labeling through photoactivation or photo-uncaging. Alternatively, introduction of additional chemical agents can be used to initiate a reaction which activates the probe.

### Photoaffinity-Based Probes

A well-established strategy in inducible probe design is to incorporate a photoactivatable cross-linker. A variety of these photoaffinity-based probes have been reviewed previously.^19−24^

As photoaffinity-based probes do not react specifically with a catalytic residue of the target protein, labeling occurs as the result of a binding interaction between the probe and protein (Figure 1d). Photoaffinity-based probes differ from activity-based probes in this respect and can be considered as effective probes for target engagement, rather than tools for detecting catalytic activity. This section highlights commonly used photoactivated cross-linking groups and recent examples of their applications.

Photoactivated groups can form highly reactive species upon irradiation with UV light. Incorporation of a photoactivatable group into a probe therefore allows an inducible covalent bond to be formed between the reactive species of the probe and a proximal residue of the target protein. This is a well-established strategy which has proven particularly useful for targeting enzymes which do not have a nucleophilic catalytic residue in their active site, such as metalloenzymes, kinases, and histone deacetylases.^18,19,25,26^

While a vast array of photoactivatable groups have been reported,^20^ a smaller subset have become popular in protein profiling as they exhibit suitable reactivity and bioorthogonality. Significant damage to the protein structure is known to occur from UV radiation <300 nm; therefore it is preferable for the wavelength of photoactivation to be >300 nm.^27^ Additionally, the half-life of the reactive intermediate should be shorter than the half-life of dissociation of the protein–probe complex, in order to avoid excess off-target labeling.^22^ Aryl azides,^28^ benzophenones,^18,25,26,29^ and diazirines^24,30−33^ have emerged as the most frequently applied photoactivatable cross-linkers for photoaffinity-based probes and target engagement.^34−36^

**Figure 2:**
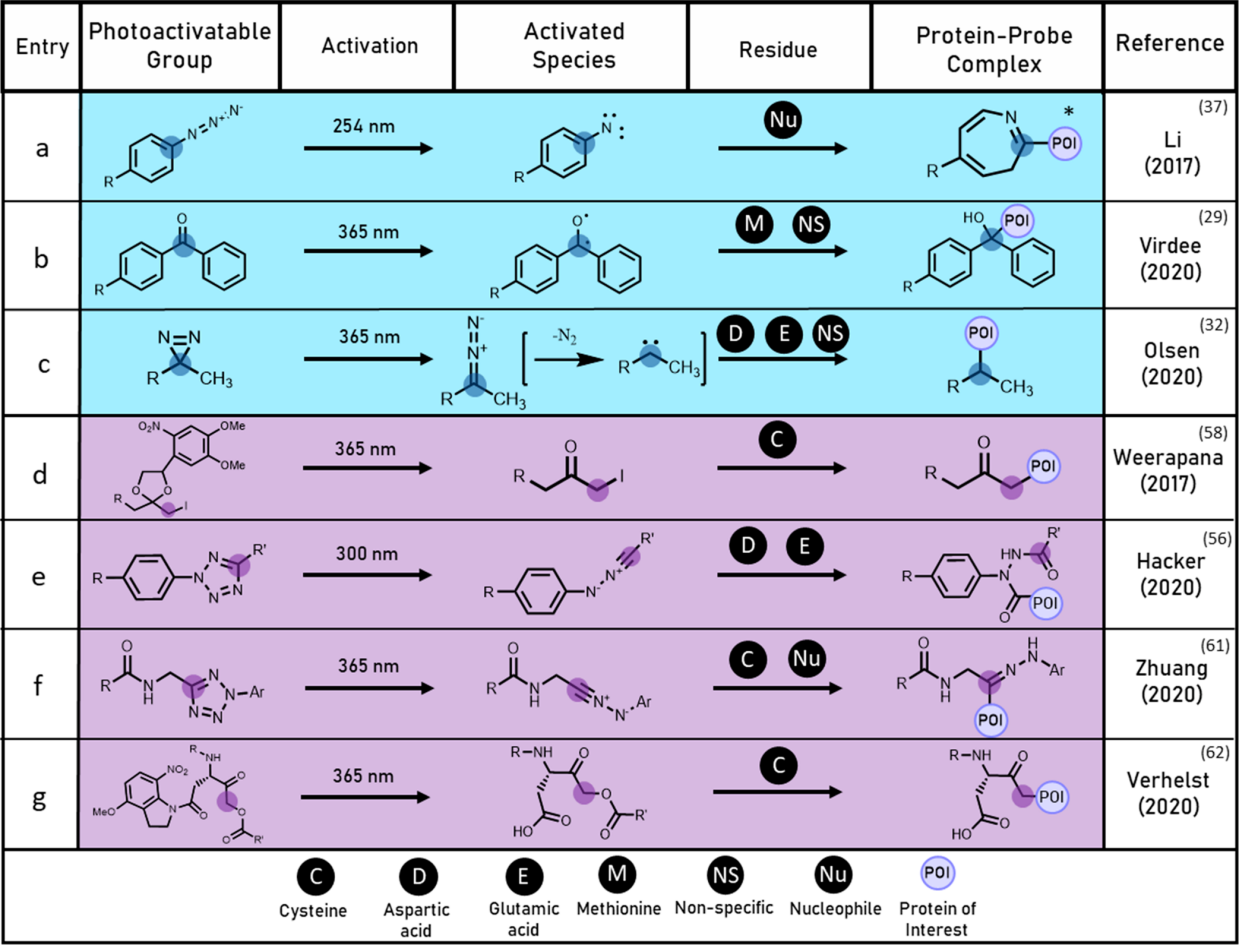
Table of photoactivated and photocaged probes indicating mode of activation, target residue, and resultant protein–probe complex given as described in the denoted reference.

Historically, aryl azides were employed as photoactivatable groups due to their convenient preparation and commercial availability. A contemporary example of this photoactivatable group was described by Li and co-workers in 2017.^37^ A diubiquitin-based probe incorporating an aryl azide enabled successful labeling of deubiquitinating enzymes (DUBs) in cell lysate (Figure 2a).^37^ Despite the success of aryl azide-containing probes, there are some disadvantages associated with use of this photoactivated group. Phenylazides are activated by short wavelengths of UV radiation (250–350 nm),^22^ a potential source of protein damage and hence sample degradation. Additionally, the reactive nitrene which forms upon aryl azide activation undergoes rearrangement to form a stabilized ketenimine which exhibits decreased cross-linking efficiency.^20^ As such, use of aryl azides as photoreactive groups in probes has become decreasingly popular in recent years.

By contrast, benzophenones are activated by longer wavelength UV (350–360 nm), resulting in reduced radiative damage to sample proteins.^22^ Upon photoactivation, the benzophenone carbonyl adopts a reactive diradical triplet state which can cross-link with the target protein, resulting in a tertiary alcohol protein–probe complex.^24^ The diradical species can also be quenched by reaction with water. However, the hydrated adduct undergoes rapid dehydration to return the original benzophenone, mitigating the detrimental impact of solvent quenching that can limit other photoactivatable groups.^20,24^ Due to this, benzophenones require a prolonged period of irradiation for effective activation, increasing the risk of nonspecific labeling.^38^ The steric bulk of the benzophenone group can also disrupt binding interactions with the target protein.^31^ Despite these factors, benzophenones have been used extensively as photoactivatable groups in target engagement probes for many years. While these photoactivatable groups are often applied to label proximal residues nonselectively, they have been found to be highly effective for labeling methionines.^39^ In 2020, Virdee and co-workers incorporated a benzophenone group into a stabilized E2-ubiquitin conjugate to generate an effective probe for labeling ubiquitin RING E3 ligases (Figure 2b).^29^

The most extensively used photoactivatable groups for protein profiling are diazirines and, in particular, aryl diazirines and aryl trifluoromethyl diazirines.^24,33^ The popularity of this photoactivatable group in recent literature can be explained by its stability and high reactivity upon activation. Like benzophenones, diazirines require a longer wavelength for activation (350–380 nm),^22^ while occupying a relatively small steric footprint. Their small size allows them to be incorporated into biologically active probe scaffolds with little disruption to target binding.^31,33,40^ Upon photoactivation, diazirines can release N_2_ to form reactive carbenes or rearrange to form electrophilic diazo compounds. Carbenes can readily cross-link with proximal residues in the protein binding site, while diazo intermediates are prone to attack by nucleophilic residues, predominantly aspartates and glutamates.^41,42^ Due to their high reactivity, carbenes which do not cross-link with a proximal residue can be readily quenched by environmental water.^33,43^ This diminishes overall labeling yield but also decreases nonspecific labeling.^24^

A diazirine containing inhibitor-based probe for the intracellular sensor protein NLRP3 was developed in 2020 by Robertson and co-workers.^31^ This work was notable as previous inhibitor-based NLRP3 probes featuring bulky benzophenones displayed significantly decreased target binding, while the comparable diazirine probe remained highly potent.^44,45^ Also in 2020, Olsen and co-workers reported a series of peptide-based diazirine probes (Figure 2c)^32^ where the photoactivatable group was introduced as a non-natural l-photoleucine amino acid residue. These probes were used to examine proteins that target ε-N-acyllysine post-translational modifications.

Photoactivatable groups allow for temporal and spatial control of probe activation and hence offer advantages over conventional probe warheads. However, as all these photoactivatable groups generate highly reactive radical nitrene or carbene species upon irradiation, regardless of binding to the target protein, off-target labeling and solvent quenching can limit their application. Furthermore, probes utilizing photoactivatable groups require only binding of the probe to the target protein in order for labeling to occur. As probe binding may not distinguish between the active and inactive target protein, their use is limited to the evaluation of protein–probe interactions, rather than the detection of enzymatic activity.

### Photocaged Activity-Based Probes

In addition to UV irradiation inducing formation of a reactive radical, carbene, or nitrene species, it is also possible to use UV light to induce formation of an electrophile. This “uncaging” strategy has been demonstrated to release a variety of functional molecules including chemotherapeutics,^46^ fluorophores,^47^ enzymes,^48,49^ and neurotransmitters.^50−52^ However, it has only recently been applied as a method for initiating protein profiling, possibly due to the incursion of protein and DNA damage inherent with UV radiation *in vitro*, combined with the limitations of low tissue penetrating power when translating profiling to *in vivo* models.^22,53−55^ A caged electrophile approach can overcome some of the limitations of existing probes, including poor cell permeability.^56,57^ As photocaged probes are predominantly unreactive, this approach allows for accumulation of the inducible probe in living cells at high concentrations with limited cytotoxicity.^16,58^ Here, we discuss the recent development of photocaged probes and their applications to study a variety of proteins.

In 2017, Weerapana and co-workers described a photocaged probe that, once uncaged, demonstrated efficient cysteine labeling in both HeLa cell lysate and in HeLa whole cells.^58^ The probe was optimized from a caged α-bromoketone^16^ to an α-iodoketone electrophile masked by a 4,5-dimethoxy-2-nitrophenyl photocage. Irradiation with UV light (365 nm) liberated the active electrophile from its protected ketal form (Figure 2d), which reacted readily with cysteine residues on a variety of proteins. The photocaged probe showed decreased cytotoxicity compared to the analogous and widely used cysteine reactive iodoacetamide alkyne probe^59^ and hence demonstrated greater labeling efficiency in living cells. As such, this probe provided an improved method for global evaluation of cysteine modifications in living cells.

In 2020, Hacker and co-workers used a series of 2,5-disubstitued tetrazole-based photocaged probes to residue-specifically map aspartates and glutamates in the bacterial proteome.^56^ When irradiated with UV light (∼300 nm), 2,5-disubstitued tetrazoles release an electrophilic nitrilimine warhead (Figure 2e). Acidic residues are capable of reacting with nitrilimines to form detectable protein–probe adducts via nucleophilic attack followed by an *O*,*N*-acyl shift.^56,60^ Labeling experiments were successful in both *S. aureus* and Gram-negative bacterial cells, demonstrating the methodology in challenging targets for cell permeation.^57^ Tetrazole bearing inducible probes therefore offer a potential strategy for profiling therapeutically relevant protein targets in bacteria.

A tetrazole photocage strategy was adopted by Zhuang and co-workers in 2020 to create inducible probes for DUBs.^61^ The authors replaced the electrophilic Michael acceptor warhead of their previously developed cell-permeable DUB probe with a tetrazole photocage. Irradiation at 365 nm resulted in uncaging to form a nitrilimine, which acted as an effective electrophilic trap for the active site cysteine residues of the target DUBs.^61^ Due to the inclusion of both labile cell penetrating peptides and a photocaged electrophilic warhead, the probe could undergo light induced labeling of DUBs in HeLa whole cells (Figure 2f), demonstrating the benefits of a combinatorial approach to inducible activity-based probe design.

The use of photocaged probes bearing a masked electrophile offers several advantages over conventional activity-based probes, with the capacity to infuse living cells with high concentrations of caged probe prior to spatiotemporally controlled initiation of electrophile formation.^16^ The photocaged probes described by both Weerapana and Abo^16,58^ and Hacker et al.^56^ are broadly reactive, labeling residues on a multitude of proteins. By contrast, Zhuang and co-workers demonstrated that a more broadly reactive photocaged electrophile approach could label selectively when a suitable recognition element is used.^61^ Therefore, there is excellent precedent to explore this strategy to specifically target further protein classes.

An alternative application of photocaged probes was demonstrated by Verhelst and co-workers in 2020.^62^ A series of selective caspase inhibitors were prepared bearing a nitroindoline photocage adjacent to the electrophilic warhead component of the probe (Figure 2g). The photocaged group in this approach sterically obstructs target binding rather than acting as a precursor to electrophile formation. UV irradiation cleaves the photocage, allowing effective binding and nucleophilic attack of caspases on the probe.^62^

Both the concept of caging the electrophilic warhead of the probe and the approach of sterically blocking binding with a caging group are widely applicable to target other enzyme classes. Several recently published approaches featuring photocaged methods for protein labeling could also be effectively used in inducible probe design. For example, nitrobenzyl photocages have been incorporated into unnatural amino acids capable of forming quinone methide electrophiles upon UV irradiation. Unnatural amino acid incorporation followed by electrophile activation enabled the covalent capture of proximal nucleophilic amino acid residues.^63^ The use of nitrobenzyl photocages as part of the protecting group strategy in the preparation of activity-based probes has also been reported.^64^ While the photocage was used as a conveniently cleavable protecting group for probe synthesis, the same uncaging step could potentially be performed *in situ* to enable labeling of the target protein. Inspiration can also be drawn from proximity labeling strategies such as photocatalytic ligand-directed labeling^65^ for inducible activity-based probe design. Visible light mediated oxidation of furans results in the formation of dicarbonyl Michael acceptors,^66^ which could also be exploited in an inducible activity-based probe approach.^67^ Photocaged probes offer an exciting strategy for inducible activity-based protein profiling, and there remains tremendous scope for further investigation in this area.

### Agent-Activated Probes

A relatively unexplored strategy in inducible probes is the application of secondary agents which enable probe activation *in situ*. This strategy offers an alternative method of switchable activation for protein profiling.

**Figure 3:**
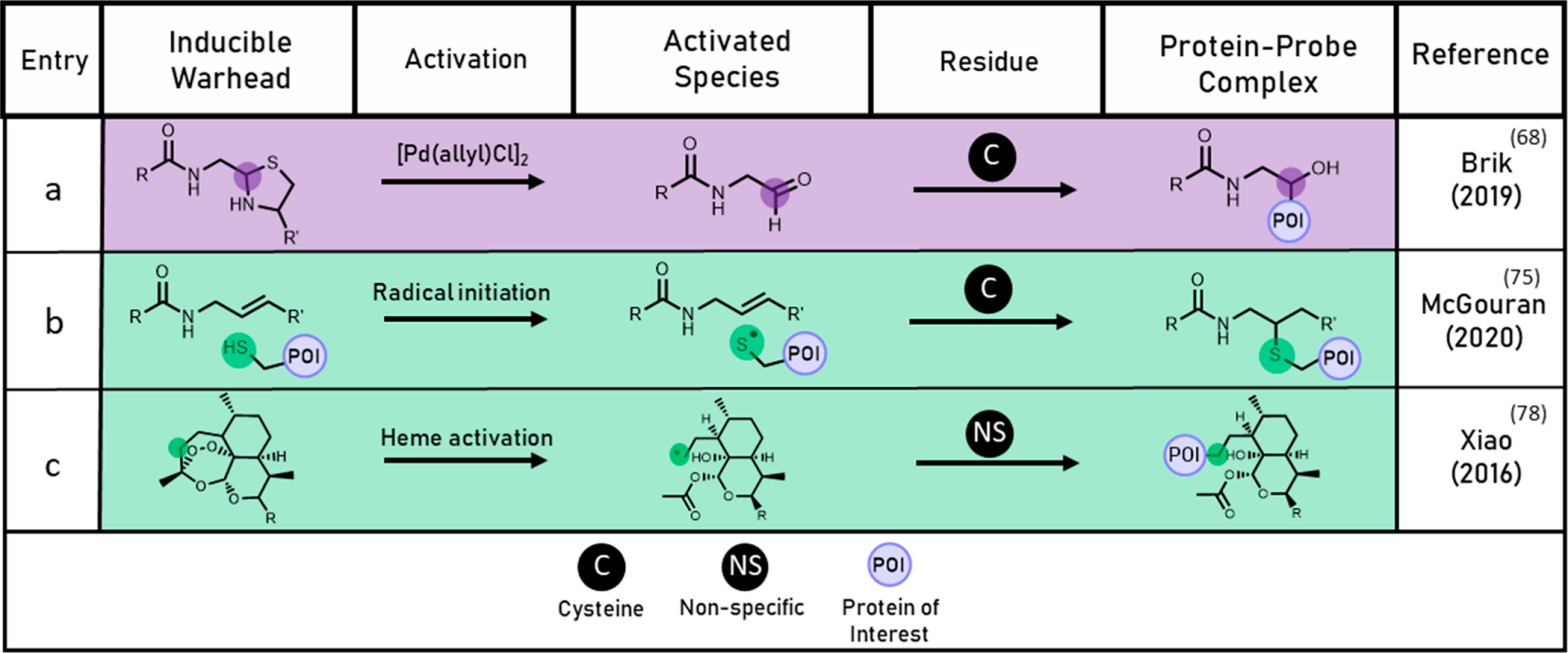
Table of agent-activated probes indicating mode of activation, target residue, and resultant protein–probe complex given as described in the denoted reference.

An agent-activated probe targeting DUBs was reported in 2019 by Brik and co-workers^68^ and depended upon the introduction of a Pd complex for probe activation. The probe was derived from a mutant ubiquitin variant, Ubv2.3, which was previously developed by Sidhu and co-workers to target USP2a.^69^ The probe incorporated a cell penetrating peptide to enable permeation of whole cells and featured a thiazolidine moiety which could be cleaved *in situ*. [Pd(allyl)Cl]_2_ was selected as the secondary agent due to its biocompatibility and low toxicity.^70−73^ Incubation of the probe in whole cells, followed by treatment with [Pd(allyl)Cl]_2_, resulted in thiazolidine cleavage and the formation of an aldehyde electrophile. It was hypothesized that the thiazolidine ring was activated for hydrolysis by an interaction between palladium and sulfur, resulting in a carbinolamine intermediate which decomposed to liberate the aldehyde warhead.^74^ This electrophilic warhead could then be attacked by the nucleophilic cysteine at the active site of USP2a to form a reversible thiohemiacetal adduct. The probe successfully labeled USP2a in whole cells (Figure 3a) validating the strategy for probe activation. This exciting approach could be further explored for the targeting of other cysteine-containing enzymes.

Another agent-activated inducible probe for DUBs was reported in 2020 by McGouran and co-workers.^75^ Whereas most inducible probes feature *in situ* activation of the probe, this approach utilized activation of the target protein. The probe consisted of a ubiquitin recognition element conjugated to a biologically inert alkene warhead (Figure 3b). Following preincubation, treatment with the radical initiator DPAP, and irradiation with UV light, a thiyl radical is formed at the active site cysteine which can undergo a thiol–ene reaction with the alkene warhead, resulting in the formation of a covalent protein–probe adduct. The probe was capable of labeling DUBs and ubiquitin conjugation machinery in HEK 293T cell lysate. This elegant strategy does not require the probe to undergo any structural changes which may affect its target binding and represents a translatable strategy for targeting other cysteine containing enzymes. The thiol–ene reaction initiated with DPAP and UV irradiation has been applied as a method for photoactivation of small molecules in human colon cancer cells, resulting in only slightly diminished cell viability,^76^ while a visible light initiated thiol–ene has also been demonstrated on purified recombinant DUBs.^77^ Further studies could help to elucidate the extent of toxicity implications of this radical initiator strategy in living systems.

In 2016, Xiao and co-workers reported an agent-activated activity-based probe derived from the natural product artemisinin.^78^ Despite decades of research into the role of artemisinin derivatives as antimalarial,^103,104^ anticancer,^101^ and anti-inflammatory^120^ therapeutics, their mechanism of action remains a subject for debate.^79^ Coordination of heme to the endoperoxide bridge of artemisinin is understood to generate reactive carbon-centered radicals which can cross-link with proximal proteins.^80,81^ Xiao and co-workers demonstrated that labeling of several glutathione-*S*-transferases with an artemisinin derived probe in cell lysate could be induced by the addition of hemin (Figure 3c), an oxidized derivative of heme. This work highlighted the potential of endoperoxide warheads for protein profiling as a non-UV dependent tool for target protein labeling.

While relatively few agent-activated probes currently exist, advances in chemical biology continue to inform new methods of probe induction. For example in 2020, Prescher and co-workers described a cyclopropenone triggered method for protein cross-linking using functionalized triaryl phosphines to create an electrophilic ketene ylide.^82^ Inverse electron-demand Diels–Alder reactions have also been demonstrated for the release of alcohol, carboxylic acid, and primary amine payloads.^83−85^ Similar strategies could be used to unmask recognition groups^62^ or to deprotect probe warheads.^58^ Such strategies appear to be readily translatable to the design of activity-based probes, opening new possibilities for probe design and expanding the repertoire of amino acid residues which can be targeted.

Although the requirement for secondary agents may complicate the application of this class of probe, particularly for *in vivo* settings, these probes provide an alternative strategy for labeling through inducible activation, and present the possibility of controlled activation without alterations in probe structure. Agent-activated probes present a promising strategy for protein profiling and may be applied further afield to target proteins that have yet to be explored.

## Endogenous Induction

Endogenous induction describes probes that are activated by an agent native to the biological sample. Endogenous activation can occur as a result of a chemical reaction such as hydrolysis, metabolism by a native enzyme in the sample, or through engagement with the target protein itself. Interaction between the endogenous activator and the inducible probe brings about a change in probe binding or reactivity, which enables labeling of the target protein. The probes discussed in this section are divided based on their mode of activation: cell-based, mechanism-based, and binding-associated.

### Cell Activated Probes

It is possible to take advantage of in-cell metabolism as a mechanism to unmask or induce activity-based probes. This strategy can offer an effective method for overcoming the low membrane permeability, which limits the use of some probes,^15^ allowing labeling experiments to be performed in more complex systems such as whole cell or *in vivo*.

**Figure 4:**
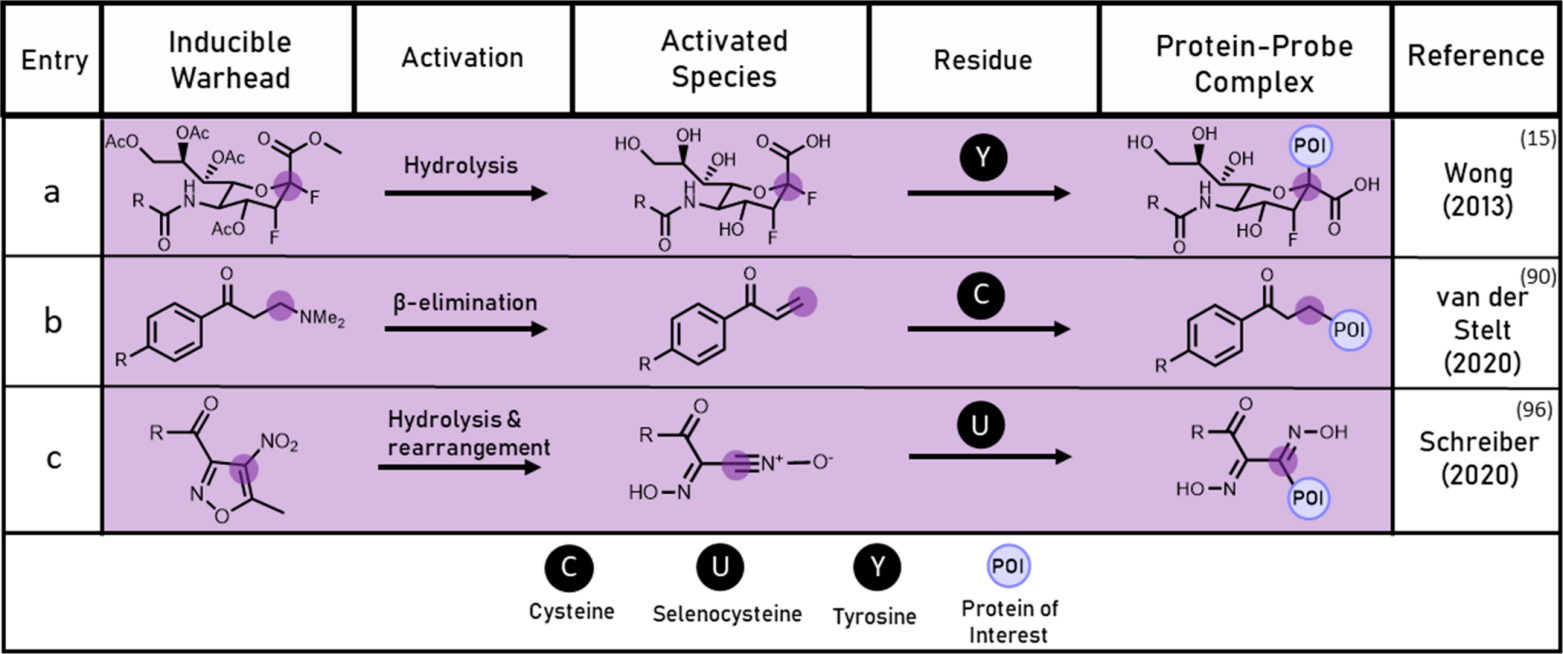
Table of cell activated probes indicating mode of activation, target residue, and resultant protein–probe complex given as described in the denoted reference.

In 2013, Wong and co-workers described an inducible activity-based probe strategy to study neuraminidases,^15^ an enzyme class implicated in diseases such as sialidosis.^86^ The probe, DFSA, showed effective labeling of the catalytic tyrosine residue of several neuraminidases in cell lysate; however, poor membrane permeability precluded application of the probe in whole cells. Peracetylation of hydroxyl groups is an established method for improving the cell permeability of saccharides,^87,88^ and the authors demonstrated that acetylation of the carbohydrate-based probe improved lipophilicity and enabled the probe to cross cell membranes. The cell-permeable probe, PDFSA, labeled a range of neuraminidases in cells once activated by *in situ* esterase hydrolysis (Figure 4a). Masking hydrophilic groups is a common prodrug strategy in medicinal chemistry and could be widely applied in the design of future cell-permeable probes.^88,89^

In 2020, van der Stelt and co-workers reported STA-55,^90^ an activity-based probe with broad-spectrum aldehyde dehydrogenase (ALDH) activity. Upregulation of ALDHs in cancer cells has been linked with chemotherapeutic resistance.^91^ STA-55 featured a Mannich base motif which unmasks *in situ* to form a vinyl ketone electrophile (Figure 4b). This surprising phenomenon was initially observed in an ALDH inhibitor screen by Hurley and co-workers^92^ and has been retrospectively discovered in other enzyme inhibitors.^93^ In both inhibitor and probe examples, the vinyl ketone generated *in situ* can label the catalytic cysteine residue of ALDHs through Michael addition. STA-55 demonstrated good permeability and successfully enriched several ALDHs present in lung cancer cells. The targeting of nucleophilic cysteine residues with an electrophilic warhead is a popular approach in activity-based protein profiling,^3,94,95^ hence there is significant scope for applying this Mannich base approach to the design of other inducible probes. Additionally, the hydrophilic amine in the inactivate form may improve the aqueous solubility of more lipophilic probes, facilitating their use in biological settings.

An inducible probe featuring a similarly noteworthy mechanism of action was described in 2020 by Schreiber and co-workers.^96^ Glutathione peroxidase 4 (GPX4) is of therapeutic significance, as the enzyme upregulated in several drug-resistant cancer variants.^97^ The screening hit ML210 was observed to inhibit GPX4 in an irreversible manner, despite bearing no covalently reactive group. Investigation with a probe analogue, ML210-yne, showed the probe to have bound to GPX4 via a selenohydroximate bridge (Figure 4c) in melanoma cells. The authors reasoned that the nitroisoxazole moiety of ML210-yne must undergo hydrolytic ring-opening and rearrangement to unmask a nitrile oxide electrophile. This nitrile oxide electrophile could then be attacked by the catalytic selenocysteine residue of GPX4.

In the course of their work, Schreiber and co-workers also demonstrated the suitability of ML210 for labeling a cysteine mutant of GPX4, highlighting the potential of using nitrile oxides for labeling proteins with active site cysteines. Nitroisoxazoles are the latest in a series of compounds, including furoxans^98^ and nitroalkanes,^99^ to feature embedded nitro substituents as masked electrophiles. Examination of other commonly metabolized functional groups may help identify yet more classes of masked electrophilic warheads for application in inducible activity-based probes.

In each of these examples, the *in situ* activation of activity-based probes allowed whole cell protein profiling.^100^ These publications demonstrated different approaches to probe design, masking either the recognition element or the warhead. Synthesis of these masked probes afforded activity-based probes with more favorable properties than their unmasked analogues. Cell-based activation of inducible probes is a powerful protein profiling strategy, with broad scope for application to other protein targets. As available metabolic pathways are better understood, new uncaging strategies for cell activated probes are likely to emerge.

### Mechanism-Based Probes

Mechanism-based probes are a well-established approach to activity-based protein profiling.^101^ These probes feature a substrate motif which can be recognized and processed by the target protein. This results in the release of a reactive electrophile, which can undergo attack from a nucleophilic residue within the active site, resulting in labeling of the target protein.^102^

The most common class of mechanism-based probes form a quinone methide electrophile upon enzymatic activation.^103^ Quinone methide mechanism-based probes have been employed for labeling of phosphatases,^104^ glycosidases,^103,105^ β-lactamases,^106^ and sulfatases.^107^ They have been used for protein profiling *in vivo* including several studies in mouse models.^108−110^ Induction occurs upon the enzymatic cleavage of an oxygen–heteroatom or oxygen–carbon bond, analogous to the turnover of the natural substrate. The resulting phenolate undergoes elimination of a conjugated leaving group, and an electrophilic quinone methide is formed. This electrophilic warhead may then be attacked by a nucleophilic residue in the enzyme active site to result in covalent capture of the protein. However, the lack of an affinity motif in the resulting quinone methide can lead to diffusion from the active site, and other proteins may be labeled nonspecifically.

**Figure 5:**
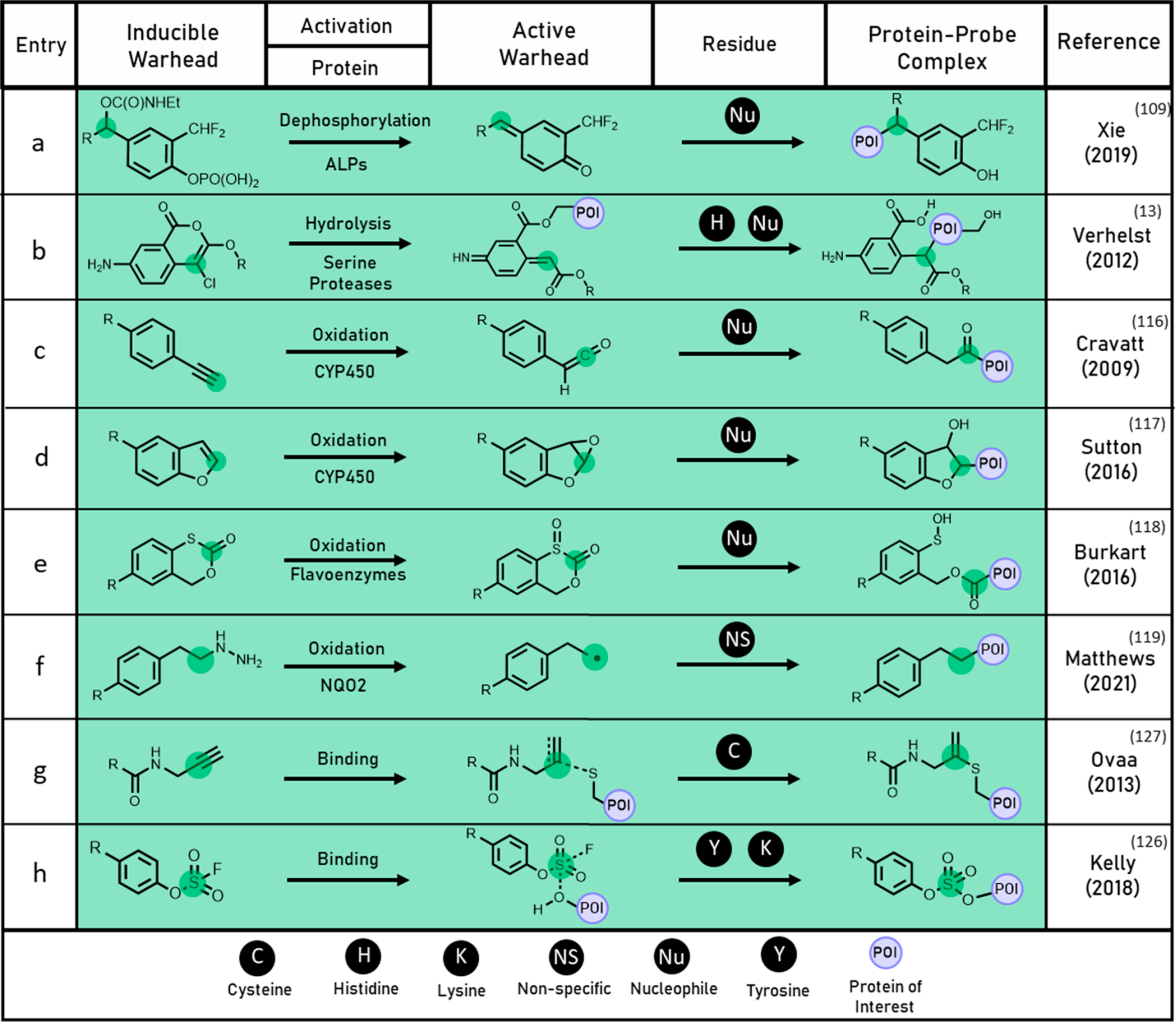
Table of mechanism-based probes and binding-associated activation of latent electrophilic probes indicating mode of activation by a protein of interest, activated species, and resultant protein–probe complex described in the denoted reference.

Among quinone methide precursors, there is variation in the aromatic substitution and leaving groups utilized. In 2019, Xie and co-workers^109^ investigated structure–activity relationships for a range of mechanism-based probes for alkaline phosphatases (ALP), a biomarker of several diseases including hepatitis.^111^ It was discovered that incorporation of self-immolative carbamate groups resulted in greater target labeling than fluoride leaving groups in the *para* benzylic position. Furthermore, multifunctional mechanism-based probes featuring both *ortho* and *para* benzylic leaving groups resulted in the highest labeling sensitivity of all probes tested in HeLa whole cells (Figure 5a). It was reasoned that the efficacy of this multifunctional probe, ALP-6, resulted from its ability to offer multiple pathways to protein labeling upon quinone methide formation. When an ALP-6 analogue was functionalized with an IR fluorescent dye, competitive phosphatase labeling in HeLa tumor xenograft mice could be demonstrated.^109^

Although a multitude of quinone methide probes exist in the literature, the efficiency of this strategy remains limited by off-target labeling.^112^ Intermediates with high affinity are retained within the active site long enough to covalently label the protein. However, low affinity or less reactive intermediates can diffuse from the active site and contribute to off-target labeling.^104^ The exception is a small class of chloroisocoumarin probes originally investigated by Verhelst and co-workers which retain the quinone methide formed within the active site of serine proteases and serine hydrolases via an intermediate ester (Figure 5b).^13,113^ However, the majority of probes lack binding affinity to prolong residence time within the active site. An exciting area for advancement therefore would be the incorporation of additional target recognition motifs which could decrease the rate of quinone methide dissociation.

Several classes of mechanism-based probes have been developed for labeling oxidative enzymes. Initial strategies focused on the cytochrome P450 (CYP) family that regulate oxidative metabolism.^114^ Cravatt and co-workers have demonstrated that metabolic oxidation of alkynes can form ketene (Figure 5c) and α,β-unsaturated ketone electrophiles,^115,116^ resulting in the labeling of a range of CYP enzymes. In 2016, Sutton and co-workers^117^ extended this work by examining 8-hydroxypsoralen analogues that underwent CYP oxidation to form electrophilic furan epoxides (Figure 5d). The benzofuran probe JDS-14 was found to effectively inhibit CYP3A4 in enzymatic assays, and labeling of the overexpressed enzyme in cell lysate was also demonstrated. JDS-14 showed NADPH-dependent labeling of several other overexpressed CYPs, validating benzofurans as an additional class of oxidatively activated probes.

Burkart and co-workers described an oxidizable probe for labeling aerobic flavin dependent enzymes (flavoenzymes), catalysts involved in the biosynthesis of several natural products.^118^ Oxidation of 1,3-oxathiin-2-one probes to give acyl sulfoxide electrophiles enabled fluorescent labeling of several purified flavoenzymes (Figure 5e), and moderate reactivity was demonstrated in *E. coli* whole cells.

In 2021, Matthews and co-workers developed a novel class of organohydrazine mechanism-based probes.^119^ Previous studies had demonstrated that organohydrazine probes could be used as nucleophiles for reverse polarity protein profiling;^120^ however detection of some unexplained adducts suggested a secondary labeling pathway. Examination revealed that enzymes which bound oxidizing cofactors at their active sites could oxidize organohydrazine probes to form a carbon centered radical for covalent protein–probe cross-linking (Figure 5f). The reaction of the probe with the enzyme NQO2 was confirmed by mass spectrometry, while X-ray crystallography confirmed that labeling occurred immediately adjacent to the binding pocket of oxidizing cofactor FAD. Organohydrazine probes showed competitive and specific labeling of cofactor bearing active enzymes, representing one of the most broadly applicable classes of oxidizable mechanism-based probes yet reported.

Applications of oxidatively activated probes are also hampered by diffusion enabled off-target labeling. While a variety of inducible warheads can be formed through enzymatic oxidation, mechanism-based probes of this nature have appeared only sparsely in the literature, which may reflect the greater applicability of quinone methides for probing several enzyme classes of therapeutic significance.^12,104^ However, there is significant scope to apply the principles demonstrated by the examples above to expand our toolbox of ways to study oxidative enzymes.

### Binding-Associated Activation of Activity-Based Probes

A recurring theme in activity-based probe design is the tuning of highly reactive, nonspecific electrophiles to create warheads with greater target or residue specificity.^121^ This tuning reduces probe electrophilicity and can produce warheads of such low reactivity that they are considered almost inert.^14^ These “latent” electrophiles do not undergo a change in chemical structure prior to labeling and can therefore only be subtly differentiated from broadly electrophilic warheads or warheads whose reactivities are augmented via protonation in the active site.^17,121−123^ As a result, latent electrophiles are at the boundary of what would be considered as an inducible probe. Latent electrophiles must be chemically stable in the presence of water and generic nucleophiles,^14^ and their reactivity should be abolished if the target protein has been denatured.^124^ Labeling can only occur when binding of the probe to the protein places the latent electrophile in a poise that facilitates nucleophilic attack by an active site residue.^125^ Protein binding interactions stabilize the intermediate which forms upon nucleophilic attack, resulting in enhanced reactivity of the electrophile following active site binding. This strategy, due to the requirement for precise positioning of the electrophile before probe labeling can occur, affords excellent selectivity and specificity in probe labeling.^124−126^

A novel latent electrophile was described by Ovaa and co-workers in 2013.^127^ The authors discovered that Ub-Prg, a polypeptide probe bearing a terminal alkyne, could bind covalently to a range of DUBs. Terminal alkynes are generally understood to be inert under physiological conditions and are commonly used as bioorthogonal ligation handles.^128^ However, alkyne bearing probes were found to covalently label the catalytic cysteine residue of DUBs (Figure 5g). Investigation revealed the formation of a vinyl thioether bridge, resulting from Markovnikov hydrothiolation of the terminal alkyne. This mechanism of labeling was unexpected due to the formation of an unstable vinylic anion intermediate. However, concurrent studies by Mootz and co-workers^129^ on related cysteine proteases highlighted a stabilizing oxyanion hole formed by neighboring amide protons.^130^ This stabilizing region is believed to reduce the activation energy of thiol addition, allowing labeling to occur. Ub-Prg exhibited equivalent reactivity and superior selectivity for a range of DUBs over the known Michael acceptor probe Ub-VME.^127^ Ovaa and co-workers have demonstrated broader applications of terminal alkyne warheads, including the development of small molecule irreversible inhibitors of cathepsin K.^131^

Sulfonyl (VI) fluoride exchange (SuFEx) has also emerged as a valuable labeling strategy.^122^ Recent advances^132^ have enabled the convenient preparation of novel libraries of arylfluorosulfate latent electrophiles, which are lower reactivity analogues of sulfonyl fluorides.^14^ In 2018, Kelly and co-workers screened three structurally distinct arylfluorosulfate activity-based probes to measure labeling in HEK 293T cell lysate.^125^ Each probe labeled a small number of functionally diverse active enzymes and minimal cross-reactivity was observed between the probes. Proteomic analysis and X-ray crystallography revealed that SuFEx labeling of these proteins occurred at tyrosine or lysine residues within the binding pockets of active enzymes (Figure 5h). Labeling was found to require proximal cationic lysine and arginine residues, indicating an important role in the stabilization of the fluoride leaving group through H-bond donation.^125^ A further study utilizing sulfuramidimidoyl fluorides for SuFEx labeling was later published by Kelly and co-workers in 2020.^133^

The valuable discovery of both arylfluorosulfate and alkyne warhead probes has instigated campaigns in covalent inhibitor drug discovery.^124,131^ There is however room for further exploration of the scope of this inducible electrophile strategy for probe design. Meticulous observation and rational design have major roles to play in identifying further examples of binding-activated electrophiles. It remains to be seen which functional groups may yet demonstrate electrophilic activity and to what extent existing electrophiles can be re-engineered. Although this class of probe lacks control over their activation, binding-associated activation represents a highpoint in selective probe design. The simplicity and elegance of this mode of activation ensures highly specific labeling and mimics the tuned enzyme–substrate reactivity native to biological systems.

## Conclusion

The emergence of inducible activity-based probes has led to an increased ability to sample active enzymes in more diverse mediums and with a greater degree of spatial and temporal control. An extensive toolbox of bioorthogonal reactions has been built up over the past two decades,^4,134^ offering new opportunities in inducible activity-based probe design. As further strategies in probe design and activation are devised, new discoveries and applications can be anticipated.

Inducible probes which are cell-permeable enable the characterization of novel enzymatic activity or multiprotein complexes which may not be detected in cell lysate.^100^ Induced activation has the potential for mediating probe toxicity in labeling experiments.^58^ While *in vivo* labeling experiments using mechanism-based probes have previously been demonstrated,^108−110^ application of inducible probes in conjunction with bioorthogonal agents could provide a more controlled method of protein profiling in cell and animal models. Unmasking strategies developed for *in vivo* probe activation could rationally be translated into a method for release of covalent drugs and vice versa.

Several applications of bioorthogonal photolabile groups have been reported,^52^ including PROTACs,^135^ unnatural amino acids,^136^ and imaging agents.^137^ Their use in probe design is also becoming increasingly popular, but there remain opportunities to apply greater finesse in photoactivation strategy.

The exploration of novel reactive groups for covalent binding of less commonly targeted residues such as thiophosphorodichloridate reagents for histidine labeling,^138^*N*-oxyl radicals for tryptophan labeling^139^ and hypervalent iodine reagents for methionine labeling^140^ will broaden the scope of protein profiling and enable the characterization of less well understood enzymes.^56,96^ The development and profiling of new residue selective electrophiles^141,142^ enhance the ability to perform protein profiling in more complex systems and will contribute to a more comprehensive understanding of the activity of these enzymes and their biological role in the greater lifecycle of the cell.

Inducible activity-based probe designs and applications will continue to be informed and enriched by advancements in chemical biology research. Innovations such as those discussed here can inspire novel masking and activation strategies that expand the scope of activity-based protein profiling tools. The next generation of inducible probes has the potential to capture a more complete picture of enzymatic activity, with ever greater control and selectivity in probe-enzyme adduct formation.

## Abbreviations

ALDH: aldehyde dehydrogenase
ALP: alkaline phosphatase
CYP: cytochrome P450
DUBs: deubiquitinating enzymes
E2: ubiquitin-conjugating enzyme
GPX4: glutathione peroxidase 4
HEK: human embryonic kidney cell line
HeLa: Henrietta Lacks immortal cancer cell line
IR: infrared
MBP: mechanism-based probe
NLRP3: NLR family pyrin domain containing 3
NQO2: N-ribosyldihydronicotinamide quinone reductase 2
POI: protein of interest
Prg: propargylamine
PROTAC: proteolysis-targeting chimera
RING E3: really interesting new gene ubiquitin ligase
SuFEx: sulfonyl fluoride exchange
Ub: ubiquitin
UV: ultraviolet

## Keywords

**Activity-based probes**: Chemical tools for profiling active enzymes in biological samples. Activity-based probes generally consist of three key components: a warhead, a linker/recognition element, and a reporter tag.
**Activity-based protein profiling**: A powerful proteomic technique that involves the use of small molecule probes to characterize enzyme activity in complex biological systems.
**Inducible activity-based probes**: Probes that are introduced into biological samples in an unreactive form and require *in situ* conversion to their active form before labeling can occur.
**Chemical proteomics**: The use of small molecule probes to understand protein function. The major goal of chemical proteomics is the identification of protein binding partners or targets of small molecule agonism or antagonism.
**Target identification**: Biologically active molecules often act via the binding to/inhibition of direct molecular targets such as nucleic acids or proteins. Target identification is the process of determining the targets of such biologically active molecules.
**Endogenous induction**: Probes that are capable of endogenous induction are converted from their inactive to active forms by an agent found endogenously in the biological sample.
**Exogenous induction**: Probes that utilize exogenous induction are converted to their active form by an outside stimulus such as UV light or a secondary agent.
**Spatiotemporal control**: Regulation of both when and where a probe is activated in a biological sample.

## References

[ref1] Sanman L. E.; Bogyo M. Annu. Rev. Biochem. 2014, 83, 249–273. 10.1146/annurev-biochem-060713-035352. 24905783

[ref2] Cravatt B. F.; Wright A. T.; Kozarich J. W. Annu. Rev. Biochem. 2008, 77, 383–414. 10.1146/annurev.biochem.75.101304.124125. 18366325

[ref3] Panyain N.; Kennedy C. R.; Howard R. T.; Tate E. W. in Target Discovery and Validation: Methods and Strategies for Drug Discovery, 1st ed.; Plowright A. T., Ed.; Wiley-VCHVerlag GmbH& Co. KGaA, 2019; pp 51–95.

[ref4] Row R. D.; Prescher J. A. Acc. Chem. Res. 2018, 51, 1073–1081. 10.1021/acs.accounts.7b00606. 29727171 PMC6190717

[ref5] Shieh P.; Bertozzi C. R. Org. Biomol. Chem. 2014, 12, 9307–9320. 10.1039/C4OB01632G. 25315039 PMC4259128

[ref6] Heiss T. K.; Dorn R. S.; Prescher J. A. Chem. Rev. 2021, 121, 6802–6849. 10.1021/acs.chemrev.1c00014. 34101453 PMC10064493

[ref7] Handula M.; Chen K. T.; Seimbille Y. Molecules 2021, 26, 4640 10.3390/molecules26154640. 34361793 PMC8347371

[ref8] Pichler C. M.; Krysiak J.; Breinbauer R. Bioorg. Med. Chem. 2016, 24, 3291–3303. 10.1016/j.bmc.2016.03.050. 27085673

[ref9] Niphakis M. J.; Cravatt B. F. Annu. Rev. Biochem. 2014, 83, 341–377. 10.1146/annurev-biochem-060713-035708. 24905785

[ref10] Borodovsky A.; Ovaa H.; Kolli N.; Gan-Erdene T.; Wilkinson K. D.; Ploegh H. L.; Kessler B. M. Chem. Biol. 2002, 9, 1149–1159. 10.1016/S1074-5521(02)00248-X. 12401499

[ref11] Taylor N. C.; McGouran J. F. Front. Chem. 2020, 7, 914 10.3389/fchem.2019.00914. 31998698 PMC6966607

[ref12] Wu L.; Armstrong Z.; Schröder S. P.; de Boer C.; Artola M.; Aerts J. M.; Overkleeft H. S.; Davies G. J. Curr. Opin. Chem. Biol. 2019, 53, 25–36. 10.1016/j.cbpa.2019.05.030. 31419756

[ref13] Haedke U.; Götz M.; Baer P.; Verhelst S. H. L. Bioorg. Med. Chem. 2012, 20, 633–640. 10.1016/j.bmc.2011.03.014. 21454080

[ref14] Martín-Gago P.; Olsen C. A. Angew. Chem., Int. Ed. 2019, 58, 957–966. 10.1002/anie.201806037. PMC6518939 30024079

[ref15] Tsai C. S.; Yen H. Y.; Lin M. I.; Tsai T. I.; Wang S. Y.; Huang W. I.; Hsu T. L.; Cheng Y. S. E.; Fang J. M.; Wong C. H. Proc. Natl. Acad. Sci. U. S. A. 2013, 110, 2466–2471. 10.1073/pnas.1222183110. 23359711 PMC3574955

[ref16] Abo M.; Weerapana E. J. Am. Chem. Soc. 2015, 137, 7087–7090. 10.1021/jacs.5b04350. 26020833

[ref17] Ward J. A.; Pinto-Fernandez A.; Cornelissen L.; Bonham S.; Díaz-Sáez L.; Riant O.; Huber K. V. M.; Kessler B. M.; Feron O.; Tate E. W. J. Med. Chem. 2020, 63, 3756–3762. 10.1021/acs.jmedchem.0c00144. 32109059 PMC7152998

[ref18] Saghatelian A.; Jessani N.; Joseph A.; Humphrey M.; Cravatt B. F. Proc. Natl. Acad. Sci. U. S. A. 2004, 101, 10000–10005. 10.1073/pnas.0402784101. 15220480 PMC454150

[ref19] Geurink P. P.; Prely L. M.; van der Marel G. A.; Bischoff R.; Overkleeft H. S. Top. Curr. Chem. 2011, 324, 85–114. 10.1007/128_2011_286. 22028098

[ref20] Holland J. P.; Gut M.; Klingler S.; Fay R.; Guillou A. Chem. - Eur. J. 2020, 26, 33–48. 10.1002/chem.201904059. 31599057

[ref21] Murale D. P.; Hong S. C.; Haque M. M.; Lee J. S. Proteome Sci. 2016, 15, 1–34. 10.1186/s12953-017-0123-3. 28652856 PMC5483283

[ref22] Wang J.; Chen Q.; Shan Y.; Pan X.; Zhang J. TrAC, Trends Anal. Chem. 2019, 115, 110–120. 10.1016/j.trac.2019.03.028.

[ref23] Smith E.; Collins I. Future Med. Chem. 2015, 7, 159–183. 10.4155/fmc.14.152. 25686004 PMC4413435

[ref24] Dubinsky L.; Krom B. P.; Meijler M. M. Bioorg. Med. Chem. 2012, 20, 554–570. 10.1016/j.bmc.2011.06.066. 21778062

[ref25] Sieber S. A.; Niessen S.; Hoover H. S.; Cravatt B. F. Nat. Chem. Biol. 2006, 2, 274–281. 10.1038/nchembio781. 16565715 PMC1538544

[ref26] Salisbury C. M.; Cravatt B. F. J. Am. Chem. Soc. 2008, 130, 2184–2194. 10.1021/ja074138u. 18217751

[ref27] Davies M. J. Biochem. Biophys. Res. Commun. 2003, 305, 761–770. 10.1016/S0006-291X(03)00817-9. 12763058

[ref28] David A.; Steer D.; Bregant S.; Devel L.; Makaritis A.; Beau F.; Yiotakis A.; Dive V. Angew. Chem., Int. Ed. 2007, 46, 3275–3277. 10.1002/anie.200604408. 17387672

[ref29] Mathur S.; Fletcher A. J.; Branigan E.; Hay R. T.; Virdee S. Cell Chem. Biol. 2020, 27, 74–82. 10.1016/j.chembiol.2019.11.013. 31859248 PMC6963778

[ref30] Grant E. K.; Fallon D. J.; Hann M. M.; Fantom K. G. M.; Quinn C.; Zappacosta F.; Annan R. S.; Chung C. wa; Bamborough P.; Dixon D. P.; et al. Angew. Chem., Int. Ed. 2020, 59, 21096–21105. 10.1002/anie.202008361. 32745361

[ref31] Hill J. R.; Coll R. C.; Schroder K.; Robertson A. A. B. Tetrahedron Lett. 2020, 61, 151849 10.1016/j.tetlet.2020.151849.

[ref32] Bæk M.; Martín-Gago P.; Laursen J. S.; Madsen J. L. H.; Chakladar S.; Olsen C. A. Chem. - Eur. J. 2020, 26, 3862–3869. 10.1002/chem.201905338. 31922630 PMC7154546

[ref33] Hill J. R.; Robertson A. A. B. J. Med. Chem. 2018, 61, 6945–6963. 10.1021/acs.jmedchem.7b01561. 29683660

[ref34] Kleiner P.; Heydenreuter W.; Stahl M.; Korotkov V. S.; Sieber S. A. Angew. Chem., Int. Ed. 2017, 56, 1396–1401. 10.1002/anie.201605993. 27981680

[ref35] Park H.; Koo J. Y.; Srikanth Y. V. V.; Oh S.; Lee J.; Park J.; Park S. B. Chem. Commun. 2016, 52, 5828–5831. 10.1039/C6CC01426G. 27043101

[ref36] Bush J. T.; Walport L. J.; McGouran J. F.; Leung I. K. H.; Berridge G.; van Berkel S. S.; Basak A.; Kessler B. M.; Schofield C. J. Chem. Sci. 2013, 4, 4115–4120. 10.1039/c3sc51708j.

[ref37] Tan X. D.; Pan M.; Gao S.; Zheng Y.; Shi J.; Li Y. M. Chem. Commun. 2017, 53, 10208–10211. 10.1039/C7CC05504H. 28857095

[ref38] Prestwich G. D.; Dorman G.; Elliott J. T.; Marecak D. M.; Chaudhary A. Photochem. Photobiol. 1997, 65, 222–234. 10.1111/j.1751-1097.1997.tb08548.x. 9066302

[ref39] Wittelsberger A.; Thomas B. E.; Mierke D. F.; Rosenblatt M. FEBS Lett. 2006, 580, 1872–1876. 10.1016/j.febslet.2006.02.050. 16516210

[ref40] Ichiishi N.; Moore K. P.; Wassermann A. M.; Wolkenberg S. E.; Krska S. W. ACS Med. Chem. Lett. 2019, 10, 56–61. 10.1021/acsmedchemlett.8b00403. 30655947 PMC6331167

[ref41] Iacobucci C.; Gotze M.; Piotrowski C.; Arlt C.; Rehkamp A.; Ihling C.; Hage C.; Sinz A. Anal. Chem. 2018, 90, 2805–2809. 10.1021/acs.analchem.7b04915. 29376325

[ref42] O’Brien J. G. K.; Jemas A.; Asare-Okai P. N.; am Ende C. W.; Fox J. M. Org. Lett. 2020, 22, 9415–9420. 10.1021/acs.orglett.0c02714. 33259213 PMC7802889

[ref43] Wang J.; Kubicki J.; Peng H.; Platz M. S. J. Am. Chem. Soc. 2008, 130, 6604–6609. 10.1021/ja711385t. 18433130

[ref44] Coll R. C.; Hill J. R.; Day C. J.; Zamoshnikova A.; Boucher D.; Massey N. L.; Chitty J. L.; Fraser J. A.; Jennings M. P.; Robertson A. A. B.; et al. Nat. Chem. Biol. 2019, 15, 556–559. 10.1038/s41589-019-0277-7. 31086327

[ref45] Vande Walle L.; Stowe I. B.; Sacha P.; Lee B. L.; Demon D.; Fossoul A.; Van Hauwermeiren F.; Saavedra P. H. V.; Simon P.; Subrt V.; Kostka L.; Stivala C. E.; Pham V. C.; Staben S. T.; Yamazoe S.; Konvalinka J.; Kayagaki N.; Lamkanfi M. PLoS Biol. 2019, 17, e3000354 10.1371/journal.pbio.3000354. 31525186 PMC6762198

[ref46] Bonnet S. Dalt. Trans. 2018, 47, 10330–10343. 10.1039/C8DT01585F. 29978870

[ref47] Li W. H.; Zheng G. Photochem. Photobiol. Sci. 2012, 11, 460–471. 10.1039/c2pp05342j. 22252510 PMC3677749

[ref48] Chang C. Y.; Fernandez T.; Panchal R.; Bayley H. J. Am. Chem. Soc. 1998, 120, 7661–7662. 10.1021/ja981649v.

[ref49] Arabaci G.; Guo X. C.; Beebe K. D.; Coggeshall K. M.; Pei D. J. Am. Chem. Soc. 1999, 121, 5085–5086. 10.1021/ja9906756.

[ref50] Fernandes M. J. G.; Gonçalves M. S. T.; Costa S. P. G. Tetrahedron 2007, 63, 10133–10139. 10.1016/j.tet.2007.07.107.

[ref51] Fernandes M. J. G.; Gonçalves M. S. T.; Costa S. P. G. Tetrahedron 2008, 64, 11175–11179. 10.1016/j.tet.2008.09.050.

[ref52] Klán P.; Šolomek T.; Bochet C. G.; Blanc A.; Givens R.; Rubina M.; Popik V.; Kostikov A.; Wirz J. Chem. Rev. 2013, 113, 119–191. 10.1021/cr300177k. 23256727 PMC3557858

[ref53] Mouchet N.; Adamski H.; Bouvet R.; Corre S.; Courbebaisse Y.; Watier E.; Mosser J.; Chesné C.; Galibert M. D. PLoS One 2010, 5, e10776 10.1371/journal.pone.0010776. 20505830 PMC2874014

[ref54] Bustamante M.; Hernandez-Ferrer C.; Tewari A.; Sarria Y.; Harrison G. I.; Puigdecanet E.; Nonell L.; Kang W.; Friedländer M. R.; Estivill X.; et al. Br. J. Dermatol. 2020, 182, 1458–1468. 10.1111/bjd.18527. 31529490 PMC7318624

[ref55] Jayakumar M. K. G.; Idris N. M.; Zhang Y. Proc. Natl. Acad. Sci. U. S. A. 2012, 109, 8483–8488. 10.1073/pnas.1114551109. 22582171 PMC3365215

[ref56] Bach K.; Beerkens B. L. H. H.; Zanon P. R. A. A.; Hacker S. M. ACS Cent. Sci. 2020, 6, 546–554. 10.1021/acscentsci.9b01268. 32342004 PMC7181327

[ref57] Zgurskaya H. I.; López C. A.; Gnanakaran S. ACS Infect. Dis. 2015, 1, 512–522. 10.1021/acsinfecdis.5b00097. 26925460 PMC4764994

[ref58] Abo M.; Bak D. W.; Weerapana E. ChemBioChem 2017, 18, 81–84. 10.1002/cbic.201600524. 27813293 PMC5209257

[ref59] Weerapana E.; Wang C.; Simon G. M.; Richter F.; Khare S.; Dillon M. B. D.; Bachovchin D. A.; Mowen K.; Baker D.; Cravatt B. F. Nature 2010, 468, 790–797. 10.1038/nature09472. 21085121 PMC3058684

[ref60] Zhao S.; Dai J.; Hu M.; Liu C.; Meng R.; Liu X.; Wang C.; Luo T. Chem. Commun. 2016, 52, 4702–4705. 10.1039/C5CC10445A. 26953773

[ref61] Gui W.; Shen S.; Zhuang Z. J. Am. Chem. Soc. 2020, 142, 19493–19501. 10.1021/jacs.9b12426. 33141564 PMC8462974

[ref62] Chakrabarty S.; Verhelst S. H. L. Cell Chem. Biol. 2020, 27, 1434–1440. 10.1016/j.chembiol.2020.08.001. 32814013

[ref63] Liu J.; Li S.; Aslam N. A.; Zheng F.; Yang B.; Cheng R.; Wang N.; Rozovsky S.; Wang P. G.; Wang Q.; et al. J. Am. Chem. Soc. 2019, 141, 9458–9462. 10.1021/jacs.9b01738. 31184146 PMC7050464

[ref64] Liu J.; Li Y.; Deol K. K.; Strieter E. R. Org. Lett. 2019, 21, 6790–6794. 10.1021/acs.orglett.9b02406. 31398045 PMC7007859

[ref65] Beard H. A.; Hauser J. R.; Walko M.; George R. M.; Wilson A. J.; Bon R. S. Commun. Chem. 2019, 2, 1–9. 10.1038/s42004-019-0235-z. PMC7610391 33763603

[ref66] Llamas E. M.; Tome J. P. C.; Rodrigues J. M. M.; Torres T.; Madder A. Org. Biomol. Chem. 2017, 15, 5402–5409. 10.1039/C7OB01269A. 28627569

[ref67] Gehringer M.; Laufer S. A. J. Med. Chem. 2019, 62, 5673–5724. 10.1021/acs.jmedchem.8b01153. 30565923

[ref68] Mann G.; Satish G.; Meledin R.; Vamisetti G. B.; Brik A. Angew. Chem., Int. Ed. 2019, 58, 13540–13549. 10.1002/anie.201906545. 31402546

[ref69] Ernst A.; Avvakumov G.; Tong J.; Fan Y.; Zhao Y.; Alberts P.; Persaud A.; Walker J. R.; Neculai A. M.; Neculai D.; et al. Science 2013, 339, 590–595. 10.1126/science.1230161. 23287719 PMC3815447

[ref70] Yang M.; Li J.; Chen P. R. Chem. Soc. Rev. 2014, 43, 6511–6526. 10.1039/C4CS00117F. 24867400

[ref71] Li J.; Yu J.; Zhao J.; Wang J.; Zheng S.; Lin S.; Chen L.; Yang M.; Jia S.; Zhang X.; et al. Nat. Chem. 2014, 6, 352–361. 10.1038/nchem.1887. 24651204

[ref72] Martínez-Calvo M.; Couceiro J. R.; Destito P.; Rodríguez J.; Mosquera J.; Mascareñas J. L. ACS Catal. 2018, 8, 6055–6061. 10.1021/acscatal.8b01606. 30018848 PMC6038097

[ref73] Wang J.; Zheng S.; Liu Y.; Zhang Z.; Lin Z.; Li J.; Zhang G.; Wang X.; Li J.; Chen P. R. J. Am. Chem. Soc. 2016, 138, 15118–15121. 10.1021/jacs.6b08933. 27797486

[ref74] Jbara M.; Maity S. K.; Seenaiah M.; Brik A. J. Am. Chem. Soc. 2016, 138, 5069–5075. 10.1021/jacs.5b13580. 27023072

[ref75] Taylor N. C.; Hessman G.; Kramer H. B.; McGouran J. F. Chem. Sci. 2020, 11, 2967–2972. 10.1039/C9SC05258E. 34122797 PMC8157568

[ref76] Sun S.; Oliveira B. L.; Jiménez-Osés G.; Bernardes G. J. L. Angew. Chem., Int. Ed. 2018, 57, 15832–15835. 10.1002/anie.201811338. PMC6391964 30300959

[ref77] Taylor N. C.; McGouran J. F. Org. Biomol. Chem. 2021, 19, 2177–2181. 10.1039/D1OB00253H. 33630007

[ref78] Zhou Y.; Li W.; Xiao Y. ACS Chem. Biol. 2016, 11, 882–888. 10.1021/acschembio.5b01043. 26854499

[ref79] Meshnick S. R.; Taylor T. E.; Kamchonwongpaisan S. Microbiol. Rev. 1996, 60, 301–315. 10.1128/mr.60.2.301-315.1996. 8801435 PMC239445

[ref80] Wei C.; Zhao C. X.; Liu S.; Zhao J. H.; Ye Z.; Wang H.; Yu S. S.; Zhang C. J. Chem. Commun. 2019, 55, 9535–9538. 10.1039/C9CC03719E. 31334508

[ref81] Wang J.; Zhang C.-J.; Chia W. N.; Loh C. C. Y.; Li Z.; Lee Y. M.; He Y.; Yuan L.-X.; Lim T. K.; Liu M.; Liew C. X.; Lee Y. Q.; Zhang J.; Lu N.; Lim C. T.; Hua Z.-C.; Liu B.; Shen H.-M.; Tan K. S. W.; Lin Q. Nat. Commun. 2015, 6, 10111 10.1038/ncomms10111. 26694030 PMC4703832

[ref82] Row R. D.; Nguyen S. S.; Ferreira A. J.; Prescher J. A. Chem. Commun. 2020, 56, 10883–10886. 10.1039/D0CC04600K. PMC7501174 32808608

[ref83] Davies S.; Oliveira B. L.; Bernardes G. J. L. Org. Biomol. Chem. 2019, 17, 5725–5730. 10.1039/C9OB01167F. 31135016

[ref84] Davies S.; Qiao L.; Oliveira B. L.; Navo C. D.; Jiménez-Osés G.; Bernardes G. J. L. ChemBioChem 2019, 20, 1541–1546. 10.1002/cbic.201900098. 30773780

[ref85] Versteegen R. M.; Rossin R.; Ten Hoeve W.; Janssen H. M.; Robillard M. S. Angew. Chem., Int. Ed. 2013, 52, 14112–14116. 10.1002/anie.201305969. 24281986

[ref86] Maurice P.; Baud S.; Bocharova O. V.; Bocharov E. V.; Kuznetsov A. S.; Kawecki C.; Bocquet O.; Romier B.; Gorisse L.; Ghirardi M.; Duca L.; Blaise S.; Martiny L.; Dauchez M.; Efremov R. G.; Debelle L. Sci. Rep. 2016, 6, 38363 10.1038/srep38363. 27917893 PMC5137157

[ref87] Sarkar A. K.; Fritz T. A.; Taylor W. H.; Esko J. D. Proc. Natl. Acad. Sci. U. S. A. 1995, 92, 3323–3327. 10.1073/pnas.92.8.3323. 7724561 PMC42158

[ref88] Xing B.; Khanamiryan A.; Rao J. J. Am. Chem. Soc. 2005, 127, 4158–4159. 10.1021/ja042829+. 15783183

[ref89] Rautio J.; Meanwell N. A.; Di L.; Hageman M. J. Nat. Rev. Drug Discovery 2018, 17, 559–587. 10.1038/nrd.2018.46. 29700501

[ref90] Koenders S. T. A.; van Rooden E. J.; van den Elst H.; Florea B. I.; Overkleeft H. S.; van der Stelt M. ChemBioChem 2020, 21, 1911–1917. 10.1002/cbic.201900771. 31985142

[ref91] Emadi A.; Jones R. J.; Brodsky R. A. Nat. Rev. Clin. Oncol. 2009, 6, 638–647. 10.1038/nrclinonc.2009.146. 19786984

[ref92] Khanna M.; Chen C. H.; Kimble-Hill A.; Parajuli B.; Perez-Miller S.; Baskaran S.; Kim J.; Dria K.; Vasiliou V.; Mochly-Rosen D.; et al. J. Biol. Chem. 2011, 286, 43486–43494. 10.1074/jbc.M111.293597. 22021038 PMC3234859

[ref93] Roman G. Eur. J. Med. Chem. 2015, 89, 743–816. 10.1016/j.ejmech.2014.10.076. 25462280 PMC7115492

[ref94] Jones L. H. ACS Med. Chem. Lett. 2018, 9, 584–586. 10.1021/acsmedchemlett.8b00276. 30034581 PMC6047027

[ref95] Berger A. B.; Vitorino P. M.; Bogyo M. Am. J. PharmacoGenomics 2004, 4, 371–381. 10.2165/00129785-200404060-00004. 15651898

[ref96] Eaton J. K.; Furst L.; Ruberto R. A.; Moosmayer D.; Hilpmann A.; Ryan M. J.; Zimmermann K.; Cai L. L.; Niehues M.; Badock V.; et al. Nat. Chem. Biol. 2020, 16, 497–506. 10.1038/s41589-020-0501-5. 32231343 PMC7251976

[ref97] Hangauer M. J.; Viswanathan V. S.; Ryan M. J.; Bole D.; Eaton J. K.; Matov A.; Galeas J.; Dhruv H. D.; Berens M. E.; Schreiber S. L.; et al. Nature 2017, 551, 247–250. 10.1038/nature24297. 29088702 PMC5933935

[ref98] Eaton J. K.; Ruberto R. A.; Kramm A.; Viswanathan V. S.; Schreiber S. L. J. Am. Chem. Soc. 2019, 141, 20407–20415. 10.1021/jacs.9b10769. 31841309

[ref99] Ray S.; Kreitler D. F.; Gulick A. M.; Murkin A. S. ACS Chem. Biol. 2018, 13, 1470–1473. 10.1021/acschembio.8b00225. 29782144 PMC6300134

[ref100] Conole D.; Mondal M.; Majmudar J. D.; Tate E. W. Front. Chem. 2019, 7, 1–7. 10.3389/fchem.2019.00876. 31921788 PMC6930156

[ref101] Yang P.; Liu K. ChemBioChem 2015, 16, 712–724. 10.1002/cbic.201402582. 25652106

[ref102] Jeffery D. A.; Bogyo M. Curr. Opin. Biotechnol. 2003, 14, 87–95. 10.1016/S0958-1669(02)00010-1. 12566007

[ref103] Janda K. D.; Lo L. C.; Lo C. H. L.; Sim M. M.; Wang R.; Wong C. H.; Lerner R. A. Science 1997, 275, 945–948. 10.1126/science.275.5302.945. 9020070

[ref104] Polaske N. W.; Kelly B. D.; Ashworth-Sharpe J.; Bieniarz C. Bioconjugate Chem. 2016, 27, 660–666. 10.1021/acs.bioconjchem.5b00652. 26731201

[ref105] Kwan D. H.; Chen H. M.; Ratananikom K.; Hancock S. M.; Watanabe Y.; Kongsaeree P. T.; Samuels A. L.; Withers S. G. Angew. Chem., Int. Ed. 2011, 50, 300–303. 10.1002/anie.201005705. 21184404

[ref106] Mao W.; Xia L.; Wang Y.; Xie H. Chem. - Asian J. 2016, 11, 3493–3497. 10.1002/asia.201601344. 27790857

[ref107] Ahmed V.; Liu Y.; Taylor S. D. ChemBioChem 2009, 10, 1457–1461. 10.1002/cbic.200900143. 19466699

[ref108] Cheng T. C.; Roffler S. R.; Tzou S. C.; Chuang K. H.; Su Y. C.; Chuang C. H.; Kao C. H.; Chen C. S.; Harn I. H.; Liu K. Y.; et al. J. Am. Chem. Soc. 2012, 134, 3103–3110. 10.1021/ja209335z. 22239495

[ref109] Song H.; Li Y.; Chen Y.; Xue C.; Xie H. Chem. - Eur. J. 2019, 25, 13994–14002. 10.1002/chem.201903458. 31506999

[ref110] Whidbey C.; Sadler N. C.; Nair R. N.; Volk R. F.; Deleon A. J.; Bramer L. M.; Fansler S. J.; Hansen J. R.; Shukla A. K.; Jansson J. K.; et al. J. Am. Chem. Soc. 2019, 141, 42–47. 10.1021/jacs.8b09668. 30541282 PMC6533105

[ref111] Agrawal S.; Dhiman R. K.; Limdi J. K. Postgrad. Med. J. 2016, 92, 223–234. 10.1136/postgradmedj-2015-133715. 26842972

[ref112] Casey G. R.; Stains C. I. Chem. - Eur. J. 2018, 24, 7810–7824. 10.1002/chem.201705194. 29338103 PMC5986605

[ref113] Vosyka O.; Vinothkumar K. R.; Wolf E. V.; Brouwer A. J.; Liskamp R. M. J.; Verhelst S. H. L. Proc. Natl. Acad. Sci. U. S. A. 2013, 110, 2472–2477. 10.1073/pnas.1215076110. 23359682 PMC3574917

[ref114] Backes W. L.; Kelley R. W. Pharmacol. Ther. 2003, 98, 221–233. 10.1016/S0163-7258(03)00031-7. 12725870

[ref115] Wright A. T.; Cravatt B. F. Chem. Biol. 2007, 14, 1043–1051. 10.1016/j.chembiol.2007.08.008. 17884636 PMC2044501

[ref116] Wright A. T.; Song J. D.; Cravatt B. F. J. Am. Chem. Soc. 2009, 131, 10692–10700. 10.1021/ja9037609. 19583257 PMC2737065

[ref117] Sellars J. D.; Skipsey M.; Sadr-ul-Shaheed; Gravell S.; Abumansour H.; Kashtl G.; Irfan J.; Khot M.; Pors K.; Patterson L. H.; Sutton C. W.; et al. ChemMedChem 2016, 11, 1122–1128. 10.1002/cmdc.201600134. 27154431

[ref118] McCulloch I. P.; La Clair J. J.; Jaremko M. J.; Burkart M. D. ChemBioChem 2016, 17, 1598–1601. 10.1002/cbic.201600275. 27271974 PMC5656434

[ref119] Lin Z.; Wang X.; Bustin K. A.; Shishikura K.; McKnight N. R.; He L.; Suciu R. M.; Hu K.; Han X.; Ahmadi M.; et al. ACS Cent. Sci. 2021, 7, 1524–1534. 10.1021/acscentsci.1c00616. 34584954 PMC8461768

[ref120] Matthews M. L.; He L.; Horning B. D.; Olson E. J.; Correia B. E.; Yates J. R.; Dawson P. E.; Cravatt B. F. Nat. Chem. 2017, 9, 234–243. 10.1038/nchem.2645. 28221344 PMC5325178

[ref121] Shannon D. A.; Weerapana E. Curr. Opin. Chem. Biol. 2015, 24, 18–26. 10.1016/j.cbpa.2014.10.021. 25461720

[ref122] Jones L. H.; Kelly J. W. RSC Med. Chem. 2020, 11, 10–17. 10.1039/C9MD00542K. 33479601 PMC7460715

[ref123] Jiang J.; Artola M.; Beenakker T. J. M.; Schröder S. P.; Petracca R.; de Boer C.; Aerts J. M. F. G.; van der Marel G. A.; Codée J. D. C.; Overkleeft H. S. Eur. J. Org. Chem. 2016, 2016, 3671–3678. 10.1002/ejoc.201600472.

[ref124] Zheng Q.; Woehl J. L.; Kitamura S.; Santos-Martins D.; Smedley C. J.; Li G.; Forli S.; Moses J. E.; Wolan D. W.; Sharpless K. B. Proc. Natl. Acad. Sci. U. S. A. 2019, 116, 18808–18814. 10.1073/pnas.1909972116. 31484779 PMC6754619

[ref125] Mortenson D. E.; Brighty G. J.; Plate L.; Bare G.; Chen W.; Li S.; Wang H.; Cravatt B. F.; Forli S.; Powers E. T.; et al. J. Am. Chem. Soc. 2018, 140, 200–210. 10.1021/jacs.7b08366. 29265822 PMC5762408

[ref126] Chen W.; Dong J.; Plate L.; Mortenson D. E.; Brighty G. J.; Li S.; Liu Y.; Galmozzi A.; Lee P. S.; Hulce J. J.; et al. J. Am. Chem. Soc. 2016, 138, 7353–7364. 10.1021/jacs.6b02960. 27191344 PMC4909538

[ref127] Ekkebus R.; Van Kasteren S. I.; Kulathu Y.; Scholten A.; Berlin I.; Geurink P. P.; De Jong A.; Goerdayal S.; Neefjes J.; Heck A. J. R.; et al. J. Am. Chem. Soc. 2013, 135, 2867–2870. 10.1021/ja309802n. 23387960 PMC3585465

[ref128] Patterson D. M.; Nazarova L. A.; Prescher J. A. ACS Chem. Biol. 2014, 9, 592–605. 10.1021/cb400828a. 24437719

[ref129] Sommer S.; Weikart N. D.; Linne U.; Mootz H. D. Bioorg. Med. Chem. 2013, 21, 2511–2517. 10.1016/j.bmc.2013.02.039. 23535560

[ref130] Arkona C.; Rademann J. Angew. Chem., Int. Ed. 2013, 52, 8210–8212. 10.1002/anie.201303544. 23794491

[ref131] Mons E.; Jansen I. D. C.; Loboda J.; van Doodewaerd B. R.; Hermans J.; Verdoes M.; van Boeckel C. A. A.; van Veelen P. A.; Turk B.; Turk D.; Ovaa H. J. Am. Chem. Soc. 2019, 141, 3507–3514. 10.1021/jacs.8b11027. 30689386 PMC6396318

[ref132] Dong J.; Krasnova L.; Finn M. G.; Sharpless K. B. Angew. Chem., Int. Ed. 2014, 53, 9430–9448. 10.1002/anie.201309399. 25112519

[ref133] Brighty G. J.; Botham R. C.; Li S.; Nelson L.; Mortenson D. E.; Li G.; Morisseau C.; Wang H.; Hammock B. D.; Sharpless K. B.; et al. Nat. Chem. 2020, 12, 906–913. 10.1038/s41557-020-0530-4. 32868886 PMC7541551

[ref134] Shieh P.; Hill M. R.; Zhang W.; Kristufek S. L.; Johnson J. A. Chem. Rev. 2021, 121, 7059–7121. 10.1021/acs.chemrev.0c01282. 33823111

[ref135] Xue G.; Wang K.; Zhou D.; Zhong H.; Pan Z. J. Am. Chem. Soc. 2019, 141, 18370–18374. 10.1021/jacs.9b06422. 31566962

[ref136] Welegedara A. P.; Adams L. A.; Huber T.; Graham B.; Otting G. Bioconjugate Chem. 2018, 29, 2257–2264. 10.1021/acs.bioconjchem.8b00254. 29874064

[ref137] Ren M.; Li Z.; Nie J.; Wang L.; Lin W. Chem. Commun. 2018, 54, 9238–9241. 10.1039/C8CC04926B. 30066708

[ref138] Jia S.; He D.; Chang C. J. J. Am. Chem. Soc. 2019, 141, 7294–7301. 10.1021/jacs.8b11912. 31017395 PMC6996876

[ref139] Seki Y.; Ishiyama T.; Sasaki D.; Abe J.; Sohma Y.; Oisaki K.; Kanai M. J. Am. Chem. Soc. 2016, 138, 10798–10801. 10.1021/jacs.6b06692. 27534812

[ref140] Taylor M. T.; Nelson J. E.; Suero M. G.; Gaunt M. J. Nature 2018, 562, 563–568. 10.1038/s41586-018-0608-y. 30323287 PMC6203954

[ref141] Vantourout J. C.; Adusumalli S. R.; Knouse K. W.; Flood D. T.; Ramirez A.; Padial N. M.; Istrate A.; Maziarz K.; Degruyter J. N.; Merchant R. R.; et al. J. Am. Chem. Soc. 2020, 142, 17236–17242. 10.1021/jacs.0c05595. 32965106 PMC8350984

[ref142] Zanon P. R. A.; Yu F.; Musacchio P. Z.; Lewald L.; Zollo M.; Krauskopf K.; Raunft P.; Maher T. E.; Cigler M.; Chang C. J.; Profiling the proteome-wide selectivity of diverse electrophiles. ChemRxiv 2021 [Preprint July 12, 2021; Accessed September 24, 2021], 10.33774/chemrxiv-2021-w7rss-v2. PMC12580327 41168333

